# Supplementary material for: In situ monitoring of molecular aggregation using circular dichroism
Source: Nat Commun. 2018 Nov 23;9:4961. doi: 10.1038/s41467-018-07299-3 (PMC6251920; doi:10.1038/s41467-018-07299-3)
Supplement: Supplementary file 2 — Supplementary Information [file 41467_2018_7299_MOESM2_ESM.docx]

**Supplementary Information:**

**In Situ Monitoring the Molecular Aggregation Using Circular Dichroism**

Zhang et al.

# Supplementary Methods

(*R*)-(-)-6,6'-Dibromo-1,1'-binaphthyl-2,2'-diol (**5**), (*R*)-(+)-3,3'-dibromo-1,1'-binaphthyl-2,2'-diol (**9**), tetrakis(triphenylphosphine) palladium (Pd(PPh_3_)_4_) and diiodomethane (CH_2_I_2_) were purchased from J&K Scientific Ltd.. 4-Bromobenzophenone (**1**) and bis(pinacolato) diboron (**3**) were bought from Energy Chemical. 1-Bromohexane (**6**), potassium acetate (KAc), titanium tetrachloride (TiCl_4_) and zinc powder (Zn) were purchased from Aladdin. 18-Crown-6 was obtained from Shanghai Chemical Reagent. Bis(triphenylphosphine) palladium dichloride (PdCl_2_(dppf)) was purchased from ABCR GmbH & Co. KG. Potassium carbonate (K_2_CO_3_), sodium sulfate, ammonium chloride, hydrochloric acid, acetone, dioxane, tetrahydrofuran (THF), dichloromethane (DCM), methanol and petroleum ether (PE) were purchased from Signopharm Chemical Reagent Co., Ltd.. AIEgen Biotech Co., Ltd. provided the tetraphenylethylene (TPE) derivative (**2**) in free. Other chemicals and reagents without specification were purchased from Sigma Aldrich. All reagents used were in reagent grade. Dioxane and THF were distilled from sodium benzophenone ketyl under nitrogen immediately prior to use.

^1^H and ^13^C NMR spectra were recorded on Bruker AVANCE III 500, Bruker AVANCE III 400 or Varian NMR 300 spectrometer in deuterated chloroform (CDCl_3_) using tetramethylsilane (TMS; *δ* = 0) as internal reference. UV-Vis spectra were acquired on a Varian CARY 100 Bio UV-visible spectrophotometer. Photoluminescence (PL) were measured by a Shimadzu RF-5301PC spectrofluorophotometer. Thermogravimetric analysis (TGA) measurements were conducted on a TA-Q50 under N_2_ atmosphere at a heating rate of 10 ^o^C/min. Relative molecular weights (*M*_w_ and *M*_n_) and polydispersity index (PDI) of the polymers were estimated by a Waters PL-GPC-50 gel permeation chromatography (GPC) system equipped with refractive index (RI) detector, using a set of monodisperse polystyrenes as calibration standards and THF as the eluent at a flow rate of 1.0 mL/min. Absolute fluorescence quantum yield was measured on Hamamatsu C11347-11Quantaurus-QY. Circular dichroism (CD) measurements in solution were recorded on a Bio-logic MOS-450 spectropolarimeter while solid-state CD ones were carried out on a Applied Photophysics Chirascan Plus spectropolarimeter. Ground-state geometries were optimized through density functional theory (DFT) with a B3LYP hybrid functional at the basis set level of 6-31G (d). All the calculations were performed using the Gaussian 09 package.

## **Supplementary Note 1 |** Synthetic routes to P-**1**, P-**2**, P-**3** and P-**4**.

## ***1,2-Bis(4-bromophenyl)-1,2-diphenylethene (2)***

The dibromo-functionalized TPE derivative was provided by AIEgen Biotech Co., Ltd..^[1]^

## ***1,2-Bis[4-(4,4,5,5-tetramethyl-1,3,2-dioxaborolan-2-yl)phenyl]-1,2-diphenylethene (4)***

Into a 250 mL two-necked round bottom flask, bis(pinacolato) diboron (**3**, 1.24 g, 4.90 mmol), potassium acetate (0.58 g, 6.12 mmol), bis(triphenylphosphine) palladium dichloride (50 mg) and **2** (1.00 g, 2.04 mmol) were added. The system was degassed and refilled with nitrogen for three times. Freshly distilled dioxane (60 mL) was then injected to dissolve the samples. The resulting mixture was heated to reflux overnight. After cooling to room temperature, the crude product was extracted with DCM, washed with deionized water and saturated brine and dried over anhydrous sodium sulfate. After filtration and solvent evaporation, the crude product was purified by silica-gel column chromatography using PE/DCM mixture (4/1, v/v) as eluent. A white solid was obtained in 85% yield. ^1^H NMR (500 MHz, CDCl_3_), *δ* (TMS, ppm): 7.61 (d, 4H), 7.15-7.04 (m, 14H), 1.37 (d, 24H).

## ***(R)-(-)-6,6'-Dibromo-2,2'-bis(hexyloxy)-1,1'-binaphthyl (7)***

Compound **7** was synthesized according to our previous published procedure.^[2]^ A viscous liquid was obtained in 82% yield. ^1^H NMR (300 MHz, CDCl_3_), *δ* (TMS, ppm): 8.00 (s, 2H), 7.83 (d, 2H), 7.41 (d, 2H), 7.27 (d, 2H), 6.98 (d, 2H), 4.00-3.86 (m, 4H), 1.44-1.34 (m, 4H), 1.07-0.87 (m, 12H), 0.77-072 (m, 6H).

## ***(R)-(+)-3,3'-dibromo-2,2'-bis(hexyloxy)-1,1'-binaphthyl (9)***

Compound **9** was synthesized according to our previous published procedure.^[2]^ A white powder was obtained in 86% yield. ^1^H NMR (300 MHz, CDCl_3_), *δ* (TMS, ppm): 8.24 (s, 2H), 7.80 (d, 2H), 7.43-7.38 (m, 2H), 7.29-7.24 (m, 2H), 7.12 (d, 2H), 3.93-3.86 (m, 2H), 3.49-3.42 (m, 2H), 1.28-1.22 (m, 4H), 1.08-0.99 (m, 4H), 0.90-0.62 (m, 14H).

## ***(R)-(-)-6,6’-Dibromo-1,1’-binaphthyl-2,2’-methylenedioxy (10)***

Into a 250 mL round bottom flask were dissolved diiodomethane (1.09 g, 4.05 mmol), K_2_CO_3_ (1.12 g, 8.11 mmol) and (*R*)-(-)-6,6'-dibromo-1,1'-binaphthyl-2,2'-diol (**5**, 600 mg, 1.35 mmol) in 100 mL acetone. The mixture was heated to reflux overnight. After cooling to room temperature, the crude product was extracted with DCM, washed with deionized water and saturated brine and dried over anhydrous sodium sulfate. After filtration and solvent evaporation, the crude product was purified by silica-gel column chromatography using PE/DCM mixture (10/1, v/v) as eluent. A white solid was obtained in 85% yield. ^1^H NMR (500 MHz, CDCl_3_), *δ* (TMS, ppm): 8.09 (s, 2H), 7.90-7.85 (d, 2H), 7.50-7.45 (d, 2H), 7.40-7.35 (d, 2H), 7.33-7.28 (d, 2H), 5.68 (s, 2H).

## ***(R)-(+)-3,3’-Dibromo-1,1’-binaphthyl-2,2’-methylenedioxy (11)***

The synthetic procedure of compound **11** was similar to compound **10** but (*R*)-(+)-3,3'-dibromo-1,1'-binaphthyl-2,2'-diol (**9**) was used instead. A white solid was obtained in 30% yield. ^1^H NMR (500 MHz, CDCl_3_), *δ* (TMS, ppm): 8.27 (s, 2H), 7.88-7.84 (d, 2H), 7.50-7.45 (m, 2H), 7.43-7.39 (d, 2H), 7.34-7.29 (m, 2H), 5.72 (s, 2H).

## ***Synthesis of polymer P-1***

Into a 50 mL two-necked round bottom flask fitted with an allihn condenser, monomer **4** (50 mg, 0.09 mmol), monomer **7** (50 g, 0.08 mmol) and a catalytic amount of tetrakis (triphenylphosphine) palladium were added. The system was degassed and refilled with nitrogen at least for three times. 10 mL freshly distilled THF followed by aqueous solution of potassium carbonate (1.38 g, 10 mL) were injected. The mixture was heated to reflux for 24 h. After cooling to room temperature, the mixture was extracted with DCM and washed with deionized water and brine for three times. The combined organic layer was evaporated under reduced pressure. The crude product was re-dissolved in 5 mL THF and the resulting solution was then added dropwise into a mixture of chloroform/methanol (250 mL, 1/5, v/v) through a cotton filter under stirring. The precipitate was allowed to stand for 2 h and then filtered through suction filtration. The resulting polymer was further washed with methanol and completely dried under vacuum at room temperature. ^[3]^ A faint yellow powder was obtained in 63% yield. ^1^H NMR (500 MHz, CDCl_3_), *δ* (TMS, ppm): 8.01 (s, 2H), 7.95-7.90 (m, 2H), 7.50-7.30 (m, 8H), 7.20-7.00 (m, 16H), 7.10-7.03 (m, 34H), 4.00-3.80 (m, 4H), 1.45-1.30 (m, 4H), 1.10-0.80 (m, 12H), 0.75-0.60 (m, 6H). ^13^C NMR (125 MHz, CDCl_3_), *δ* (TMS, ppm): 155.0, 144.1, 142.8, 141.0, 139.2, 135.7, 133.7, 132.1, 131.8, 129.7, 128.0, 126.8, 126.6, 126.5, 126.3, 125.7, 120.7, 116.4, 70.0, 31.6, 29.6, 25.6, 22.7, 14.2 *M*_w_ = 11900, PDI = 2.53.

## ***Synthesis of polymer P-2***

The synthetic procedure of P-**2** was similar to that described for polymer P-**1** by changing monomer **7** to monomer **9**. A grey powder was obtained in 65% yield. ^1^H NMR (500 MHz, CDCl_3_), *δ* (TMS, ppm): 7.95-7.75 (m, 4H), 7.55-7.45 (m, 4H), 7.40-7.35 (m, 2H), 7.20-7.00 (m, 18H), 3.35 (s, 2H), 3.07 (s, 2H), 1.00−0.40 (m, 22H). ^13^C NMR (125 MHz, CDCl_3_), *δ* (TMS, ppm): 153.5, 143.9, 142.7, 140.9, 137.3,135.2, 133.8, 131.5, 131.3, 130.7, 129.8, 128.7, 127.9, 127.7, 126.5, 126.4, 125.9, 124.7, 72.6, 31.2, 29.8, 25.2, 22.5, 14.0 [*M*_w_ = 9200, PDI = 1.87

## ***Synthesis of polymer P-3***

The synthetic procedure of P-**3** is similar to that described in polymer P-**1** by changing the monomer **7** to monomer **10**. A faint yellow powder was obtained in 70% yield. ^1^H NMR (500 MHz, CDCl_3_), *δ* (TMS, ppm): 8.20−7.80 (m, 4H), 7.65−7.35 (m, 10H), 7.30−6.90 (m, 14H), 5.80−5.60 (s, 2H). ^13^C NMR (125 MHz, CDCl3), *δ* (TMS, ppm): 151.6, 144.0, 143.3, 141.0, 138.7, 137.3, 135.5, 132.3, 131.8, 130.8, 128.2, 127.6, 126.9, 126.6, 126.2, 125.7, 125.4, 121.6, 103.2 [*M*_w_ = 14000, PDI = 2.17].

## ***Synthesis of polymer P-4***

The synthetic procedure of P-**4** was similar to that described for polymer P-**1** by changing monomer **7** to monomer **11**. A grey powder was obtained in 73% yield. ^1^H NMR (400 MHz, CDCl_3_), *δ* (TMS, ppm): 8.05-7.70 (m, 4H), 7.55-7.35 (m, 8H), 7.20-6.95 (m, 16H), 5.20-5.00 (s, 2H). ^13^C NMR (100 MHz, CDCl_3_), *δ* (TMS, ppm): 148.6, 143.7, 142.8, 141.1, 135.6, 134.0, 131.7, 131.5, 131.1, 130.3, 129.2, 128.5, 127.8, 127.4, 126.8, 126.6, 126.0, 125.4, 101.7 *M*_w_ = 4800, PDI = 2.00.

## **Supplementary Figure 1 |** ^1^H NMR spectrum of P-**1** in CDCl_3_.

## **Supplementary Figure 2 |**^13^C NMR spectrum of P-**1** in CDCl_3_.

## **Supplementary Figure 3 |** ^1^H NMR spectrum of P-**2** in CDCl_3_.

## **Supplementary Figure 4 |** ^13^C NMR spectrum of P-**2** in CDCl_3_.

## **Supplementary Figure 5 |** ^1^H NMR spectrum of P-**3** in CDCl_3_.

## **Supplementary Figure 6 |** ^13^C NMR spectrum of P-**3** in CDCl_3_.

## **Supplementary Figure 7 |** ^1^H NMR spectrum of P-**4** in CDCl_3_.

## **Supplementary Figure 8 |** ^13^C NMR spectrum of P-**4** in CDCl_3_.

## **Supplementary Table 1 |** Characterization by GPC

| Polymer | *M*_w_ | PDI |
| --- | --- | --- |
| P-**1** | 11900 | 2.53 |
| P-**2** | 9200 | 1.87 |
| P-**3** | 14000 | 2.17 |
| P-**4** | 4800 | 2.00 |

*M*_w_ = weight-average molecular weight. PDI = polydispersity index = *M*_w_/*M*_n_, where *M*_n_ was number-average molecular weight. The values were estimated by GPC in THF on the basis of a linear polystyrene calibration.

## **Supplementary Table 2 |** Cartesian coordinates of optimized P-**1** (two repeating units) in the ground state calculated by the DFT, B3LYP/6-31G(d), Gaussian 09 program.

|  | X | Y | Z |
| --- | --- | --- | --- |
| C | -12.4665 | 0.359501 | 1.228506 |
| C | -11.8835 | 0.131477 | -0.05376 |
| C | -11.2179 | 1.123116 | -0.73177 |
| C | -11.0768 | 2.429791 | -0.18282 |
| C | -11.66 | 2.671659 | 1.104758 |
| C | -12.3398 | 1.624121 | 1.776805 |
| C | -10.3861 | 3.476231 | -0.86108 |
| C | -10.2904 | 4.729255 | -0.25903 |
| C | -10.8642 | 4.968784 | 1.016485 |
| C | -11.5292 | 3.961125 | 1.675833 |
| C | -9.76636 | 3.253662 | -2.20398 |
| C | -8.40037 | 2.865306 | -2.33023 |
| C | -7.82581 | 2.673638 | -3.63002 |
| C | -8.64585 | 2.869703 | -4.76808 |
| C | -9.9646 | 3.240393 | -4.64137 |
| C | -10.5298 | 3.437571 | -3.35477 |
| C | -7.55822 | 2.650549 | -1.20184 |
| C | -6.24672 | 2.272035 | -1.3516 |
| C | -5.66308 | 2.081238 | -2.63938 |
| C | -6.46617 | 2.28683 | -3.74764 |
| C | -13.1748 | -0.72861 | 1.944117 |
| C | -4.24471 | 1.671338 | -2.77358 |
| C | -3.65399 | 0.781941 | -1.85922 |
| C | -2.32532 | 0.390008 | -1.98722 |
| C | -1.52122 | 0.872275 | -3.03349 |
| C | -2.10467 | 1.780042 | -3.93516 |
| C | -3.43671 | 2.162008 | -3.81435 |
| C | -0.08168 | 0.493167 | -3.15182 |
| C | 0.853204 | 1.643158 | -3.34956 |
| C | 0.348008 | -0.80312 | -3.08166 |
| C | -0.58821 | -1.96689 | -3.15365 |
| C | 1.78668 | -1.17075 | -2.92484 |
| C | -0.48544 | -3.02266 | -2.23048 |
| C | -1.3381 | -4.1231 | -2.30121 |
| C | -2.29875 | -4.20355 | -3.31153 |
| C | -2.39778 | -3.17373 | -4.24919 |
| C | -1.55268 | -2.06782 | -4.17071 |
| C | 1.824611 | 1.629285 | -4.36503 |
| C | 2.668237 | 2.720899 | -4.56451 |
| C | 2.560642 | 3.851522 | -3.75232 |
| C | 1.593624 | 3.884843 | -2.74542 |
| C | 0.742667 | 2.797442 | -2.55403 |
| C | 2.365087 | -2.17084 | -3.72646 |
| C | 3.696077 | -2.54201 | -3.56846 |
| C | 4.507781 | -1.94642 | -2.58687 |
| C | 3.921717 | -0.96448 | -1.76949 |
| C | 2.593834 | -0.5837 | -1.93591 |
| C | -14.2923 | -0.46428 | 2.755394 |
| C | -14.9497 | -1.48286 | 3.436637 |
| C | -14.5251 | -2.81891 | 3.336038 |
| C | -13.3986 | -3.08253 | 2.53719 |
| C | -12.7464 | -2.06438 | 1.849028 |
| C | -15.1977 | -3.91399 | 4.096243 |
| C | -14.2674 | -4.82919 | 4.82714 |
| C | -16.5558 | -4.06944 | 4.126265 |
| C | -17.2422 | -4.97912 | 5.093966 |
| C | -17.4726 | -3.34221 | 3.19598 |
| C | -14.3732 | -6.22519 | 4.706181 |
| C | -13.4796 | -7.07092 | 5.361563 |
| C | -12.4603 | -6.53962 | 6.154091 |
| C | -12.336 | -5.15409 | 6.276528 |
| C | -13.2227 | -4.30888 | 5.611289 |
| C | -18.6252 | -2.70193 | 3.684707 |
| C | -19.4981 | -2.04008 | 2.822571 |
| C | -19.2501 | -2.02322 | 1.448429 |
| C | -18.1219 | -2.67434 | 0.94563 |
| C | -17.2432 | -3.32598 | 1.809542 |
| C | -18.2419 | -5.86628 | 4.656597 |
| C | -18.9044 | -6.70005 | 5.55599 |
| C | -18.5986 | -6.64935 | 6.91766 |
| C | -17.6238 | -5.75791 | 7.370095 |
| C | -16.9533 | -4.9329 | 6.468548 |
| O | -9.6207 | 5.688642 | -0.96198 |
| C | -9.42687 | 6.97918 | -0.38824 |
| C | -8.61459 | 7.804581 | -1.37747 |
| C | -8.32266 | 9.219978 | -0.86282 |
| C | -7.51215 | 10.05901 | -1.86108 |
| C | -7.23417 | 11.50176 | -1.40776 |
| O | -11.8291 | 3.811021 | -3.16528 |
| C | -12.6858 | 3.982247 | -4.29161 |
| C | -14.06 | 4.379604 | -3.76887 |
| C | -15.0811 | 4.581796 | -4.89581 |
| C | -16.466 | 4.992748 | -4.37481 |
| C | -17.5058 | 5.275066 | -5.47158 |
| C | -6.31555 | 11.61871 | -0.18535 |
| C | -17.8942 | 4.052636 | -6.31182 |
| C | 5.925168 | -2.34606 | -2.41535 |
| C | 6.514271 | -2.40748 | -1.11742 |
| C | 7.823915 | -2.7791 | -0.93588 |
| C | 8.657956 | -3.11694 | -2.03987 |
| C | 8.077945 | -3.05466 | -3.34977 |
| C | 6.72126 | -2.66952 | -3.50015 |
| C | 10.02014 | -3.50771 | -1.88073 |
| C | 10.77346 | -3.82166 | -3.00992 |
| C | 10.20267 | -3.75166 | -4.30727 |
| C | 8.888304 | -3.3779 | -4.46529 |
| C | 10.64347 | -3.60365 | -0.52436 |
| C | 11.35866 | -2.5079 | 0.041278 |
| C | 11.949 | -2.63124 | 1.342482 |
| C | 11.79892 | -3.85415 | 2.040892 |
| C | 11.10802 | -4.90911 | 1.491029 |
| C | 10.52808 | -4.78737 | 0.20174 |
| C | 11.51672 | -1.26533 | -0.63645 |
| C | 12.20356 | -0.22262 | -0.06486 |
| C | 12.6539 | -1.53458 | 1.901272 |
| O | 12.06959 | -4.19005 | -2.79021 |
| O | 9.832302 | -5.79797 | -0.39646 |
| C | 12.89321 | -4.5626 | -3.89239 |
| C | 14.2607 | -4.94192 | -3.33937 |
| C | 15.22863 | -5.40408 | -4.43587 |
| C | 16.61376 | -5.7845 | -3.89687 |
| C | 17.58008 | -6.27013 | -4.98465 |
| C | 18.96153 | -6.64744 | -4.4402 |
| C | 9.642374 | -7.03087 | 0.293161 |
| C | 8.817336 | -7.93903 | -0.60918 |
| C | 8.55004 | -9.31121 | 0.022127 |
| C | 7.716713 | -10.2344 | -0.87626 |
| C | 7.450796 | -11.6128 | -0.25784 |
| C | 6.616604 | -12.5278 | -1.16006 |
| H | -11.9933 | -0.84564 | -0.51573 |
| H | -10.7939 | 0.917915 | -1.70932 |
| H | -12.7437 | 1.829442 | 2.765331 |
| H | -10.7835 | 5.946929 | 1.476028 |
| H | -11.9659 | 4.151825 | 2.653399 |
| H | -8.21879 | 2.722369 | -5.75733 |
| H | -10.5678 | 3.384614 | -5.53012 |
| H | -7.96809 | 2.797166 | -0.20789 |
| H | -5.62713 | 2.14005 | -0.46902 |
| H | -6.06489 | 2.122343 | -4.74493 |
| H | -4.25018 | 0.37304 | -1.04818 |
| H | -1.90129 | -0.30472 | -1.26887 |
| H | -1.50074 | 2.1948 | -4.73747 |
| H | -3.85143 | 2.877461 | -4.51923 |
| H | 0.267897 | -2.97267 | -1.44928 |
| H | -1.24913 | -4.92095 | -1.56829 |
| H | -2.95965 | -5.06422 | -3.37165 |
| H | -3.1334 | -3.23191 | -5.0473 |
| H | -1.63491 | -1.27134 | -4.9037 |
| H | 1.913584 | 0.754255 | -5.0012 |
| H | 3.40928 | 2.688877 | -5.35908 |
| H | 3.220075 | 4.7015 | -3.90672 |
| H | 1.498173 | 4.761261 | -2.10947 |
| H | -0.01586 | 2.835223 | -1.77722 |
| H | 1.757585 | -2.6662 | -4.47877 |
| H | 4.107004 | -3.32993 | -4.19363 |
| H | 4.521066 | -0.47577 | -1.00637 |
| H | 2.172517 | 0.183206 | -1.29345 |
| H | -14.6638 | 0.55336 | 2.837062 |
| H | -15.8114 | -1.24438 | 4.05222 |
| H | -13.0282 | -4.10086 | 2.459689 |
| H | -11.8701 | -2.30507 | 1.253704 |
| H | -15.1628 | -6.64524 | 4.090916 |
| H | -13.5781 | -8.14756 | 5.249012 |
| H | -11.7644 | -7.19908 | 6.666036 |
| H | -11.5434 | -4.72942 | 6.887302 |
| H | -13.1117 | -3.23185 | 5.700068 |
| H | -18.8313 | -2.72192 | 4.751214 |
| H | -20.3759 | -1.54054 | 3.224585 |
| H | -19.9339 | -1.51346 | 0.774801 |
| H | -17.926 | -2.6788 | -0.12352 |
| H | -16.3682 | -3.83054 | 1.411637 |
| H | -18.4945 | -5.90256 | 3.600563 |
| H | -19.6645 | -7.38715 | 5.192892 |
| H | -19.1203 | -7.29362 | 7.620457 |
| H | -17.3865 | -5.7007 | 8.429305 |
| H | -16.1975 | -4.24154 | 6.827953 |
| H | -10.3986 | 7.45629 | -0.19173 |
| H | -8.89512 | 6.890205 | 0.570789 |
| H | -7.67554 | 7.27561 | -1.58382 |
| H | -9.16269 | 7.851714 | -2.32726 |
| H | -9.26988 | 9.736501 | -0.64549 |
| H | -7.78413 | 9.153282 | 0.092478 |
| H | -6.55693 | 9.553402 | -2.06642 |
| H | -8.05305 | 10.08578 | -2.81757 |
| H | -6.78111 | 12.04891 | -2.24532 |
| H | -8.1889 | 12.00534 | -1.19797 |
| H | -12.2852 | 4.761121 | -4.95758 |
| H | -12.7457 | 3.046112 | -4.86664 |
| H | -14.4083 | 3.602496 | -3.07673 |
| H | -13.9576 | 5.300345 | -3.18041 |
| H | -14.7152 | 5.3529 | -5.59067 |
| H | -15.1618 | 3.655593 | -5.48123 |
| H | -16.8479 | 4.20694 | -3.7063 |
| H | -16.3544 | 5.89228 | -3.75311 |
| H | -18.4093 | 5.682301 | -4.99822 |
| H | -17.127 | 6.067597 | -6.13308 |
| H | -6.10991 | 12.66859 | 0.052747 |
| H | -6.75859 | 11.16195 | 0.706825 |
| H | -5.35295 | 11.1245 | -0.36759 |
| H | -18.6763 | 4.306673 | -7.03639 |
| H | -17.0425 | 3.6556 | -6.87537 |
| H | -18.2787 | 3.243738 | -5.67779 |
| H | 5.900533 | -2.18084 | -0.25005 |
| H | 8.238254 | -2.82652 | 0.065898 |
| H | 6.315817 | -2.60537 | -4.5072 |
| H | 10.79807 | -3.99503 | -5.17938 |
| H | 8.456888 | -3.32834 | -5.46234 |
| H | 12.24069 | -3.95428 | 3.029578 |
| H | 11.01133 | -5.8338 | 2.048046 |
| H | 11.08722 | -1.15192 | -1.62647 |
| H | 12.32456 | 0.702621 | -0.62122 |
| H | 13.06537 | -1.64676 | 2.901598 |
| H | 12.44356 | -5.41147 | -4.42883 |
| H | 12.981 | -3.72519 | -4.60059 |
| H | 14.67569 | -4.07792 | -2.80514 |
| H | 14.12657 | -5.73649 | -2.59425 |
| H | 14.79764 | -6.26692 | -4.96515 |
| H | 15.33958 | -4.60952 | -5.18878 |
| H | 17.05292 | -4.91855 | -3.38022 |
| H | 16.50325 | -6.56902 | -3.1336 |
| H | 17.13978 | -7.13644 | -5.4987 |
| H | 17.68913 | -5.48707 | -5.74863 |
| H | 19.62726 | -6.99003 | -5.24056 |
| H | 19.44206 | -5.79138 | -3.95078 |
| H | 18.88941 | -7.45323 | -3.69924 |
| H | 10.61563 | -7.49094 | 0.520672 |
| H | 9.12155 | -6.85523 | 1.246177 |
| H | 7.869592 | -7.43567 | -0.83848 |
| H | 9.34741 | -8.05825 | -1.56277 |
| H | 9.506993 | -9.80138 | 0.255633 |
| H | 8.032557 | -9.17971 | 0.983986 |
| H | 6.757281 | -9.74786 | -1.10564 |
| H | 8.230051 | -10.3621 | -1.84074 |
| H | 8.410449 | -12.0986 | -0.03026 |
| H | 6.939285 | -11.4844 | 0.706762 |
| H | 6.447116 | -13.5045 | -0.69259 |
| H | 5.635546 | -12.0862 | -1.374 |
| H | 7.117157 | -12.7006 | -2.12079 |
| C | 12.79484 | -0.33312 | 1.229019 |
| H | 13.33386 | 0.469974 | 1.686619 |

## **Supplementary Table 3 |** Cartesian coordinates of optimized P-**2** (two repeating units) in the ground state calculated by the DFT, B3LYP/6-31G(d), Gaussian 09 program.

|  | X | Y | Z |
| --- | --- | --- | --- |
| C | 6.318973 | -6.77002 | 2.367342 |
| C | 5.92011 | -5.90582 | 1.370959 |
| C | 6.497925 | -4.61132 | 1.248428 |
| C | 7.502376 | -4.22493 | 2.194029 |
| C | 7.890078 | -5.14018 | 3.210031 |
| C | 7.315322 | -6.38733 | 3.296819 |
| H | 5.864821 | -7.75465 | 2.440659 |
| H | 5.157601 | -6.20996 | 0.66172 |
| C | 6.116683 | -3.68465 | 0.223641 |
| C | 8.066151 | -2.92853 | 2.111359 |
| H | 8.65226 | -4.83257 | 3.922418 |
| H | 7.620945 | -7.07833 | 4.077765 |
| C | 7.687142 | -2.01908 | 1.141305 |
| C | 6.705459 | -2.43006 | 0.181829 |
| H | 8.793586 | -2.6314 | 2.862728 |
| C | 5.077973 | -4.0416 | -0.79426 |
| C | 5.378904 | -4.92358 | -1.88294 |
| C | 3.807007 | -3.49801 | -0.68486 |
| C | 6.666409 | -5.49755 | -2.07635 |
| C | 4.345374 | -5.24364 | -2.82195 |
| C | 2.771067 | -3.79174 | -1.62989 |
| C | 6.914687 | -6.3441 | -3.13483 |
| H | 7.457433 | -5.2622 | -1.3721 |
| C | 4.635427 | -6.11944 | -3.90315 |
| C | 3.06449 | -4.66071 | -2.66443 |
| C | 5.890438 | -6.66174 | -4.05849 |
| H | 7.905563 | -6.77193 | -3.26225 |
| H | 3.842028 | -6.35244 | -4.60977 |
| H | 2.30061 | -4.88095 | -3.40606 |
| H | 6.098976 | -7.33074 | -4.88898 |
| O | 6.304557 | -1.54446 | -0.79341 |
| O | 3.548343 | -2.62209 | 0.34556 |
| C | 7.175505 | -1.44913 | -1.93443 |
| C | 6.585684 | -0.41741 | -2.88407 |
| H | 7.247714 | -2.43381 | -2.41711 |
| H | 8.181887 | -1.15208 | -1.60898 |
| C | 7.426397 | -0.2328 | -4.15374 |
| H | 5.56656 | -0.72806 | -3.14775 |
| H | 6.497264 | 0.537255 | -2.34922 |
| C | 6.840004 | 0.8074 | -5.11687 |
| H | 8.448695 | 0.06559 | -3.87598 |
| H | 7.521682 | -1.19624 | -4.67574 |
| H | 5.819052 | 0.507666 | -5.39563 |
| H | 6.741016 | 1.770541 | -4.59449 |
| C | 2.885303 | -3.18929 | 1.489382 |
| C | 2.692136 | -2.0784 | 2.510699 |
| H | 3.503283 | -3.99936 | 1.900693 |
| H | 1.919404 | -3.61502 | 1.184878 |
| C | 1.996478 | -2.56302 | 3.788949 |
| H | 3.674662 | -1.65335 | 2.753204 |
| H | 2.106098 | -1.27518 | 2.04519 |
| C | 1.805172 | -1.45029 | 4.827564 |
| H | 1.016241 | -2.99224 | 3.531826 |
| H | 2.578969 | -3.38103 | 4.237603 |
| H | 2.786192 | -1.02685 | 5.088736 |
| H | 1.230374 | -0.6276 | 4.376874 |
| C | 7.674277 | 1.004754 | -6.38889 |
| C | 7.080682 | 2.044341 | -7.34503 |
| H | 7.772894 | 0.041895 | -6.91024 |
| H | 8.694756 | 1.30473 | -6.11014 |
| H | 7.69754 | 2.160665 | -8.24347 |
| H | 6.072809 | 1.755535 | -7.66782 |
| H | 7.003895 | 3.027227 | -6.86392 |
| C | 1.099606 | -1.91816 | 6.106655 |
| C | 0.917044 | -0.80047 | 7.138518 |
| H | 1.67266 | -2.74159 | 6.556295 |
| H | 0.11763 | -2.33884 | 5.845873 |
| H | 0.409817 | -1.16546 | 8.038952 |
| H | 1.883962 | -0.38384 | 7.446464 |
| H | 0.31844 | 0.022823 | 6.729478 |
| C | 8.272936 | -0.65237 | 1.134794 |
| C | 7.471755 | 0.494282 | 0.987663 |
| C | 9.649861 | -0.46629 | 1.343493 |
| C | 8.028615 | 1.767089 | 1.057081 |
| H | 6.405023 | 0.381725 | 0.830558 |
| C | 10.20702 | 0.80827 | 1.397036 |
| H | 10.2943 | -1.33442 | 1.453859 |
| C | 9.409872 | 1.95459 | 1.245973 |
| H | 7.382702 | 2.635741 | 0.964183 |
| H | 11.27528 | 0.919656 | 1.554175 |
| C | 9.98653 | 3.328839 | 1.343579 |
| C | 11.11129 | 3.709898 | 0.66681 |
| C | 9.23221 | 4.257724 | 2.240775 |
| C | 11.83889 | 4.982419 | 0.960079 |
| C | 11.70985 | 2.88683 | -0.42859 |
| C | 8.8386 | 3.846582 | 3.526407 |
| C | 8.844457 | 5.536352 | 1.806251 |
| C | 12.24555 | 5.829428 | -0.08633 |
| C | 12.18721 | 5.339132 | 2.274104 |
| C | 13.09147 | 2.627091 | -0.45491 |
| C | 10.92422 | 2.402046 | -1.48818 |
| C | 8.111489 | 4.69537 | 4.359492 |
| H | 9.110619 | 2.85338 | 3.872819 |
| C | 8.106868 | 6.381247 | 2.634331 |
| H | 9.123836 | 5.864646 | 0.809902 |
| C | 12.94578 | 7.00615 | 0.174316 |
| H | 12.00395 | 5.560953 | -1.11096 |
| C | 12.89802 | 6.509633 | 2.534372 |
| H | 11.89785 | 4.689316 | 3.094041 |
| C | 13.66136 | 1.879995 | -1.4847 |
| H | 13.71911 | 3.012299 | 0.343867 |
| C | 11.49557 | 1.665691 | -2.52488 |
| H | 9.85864 | 2.609323 | -1.49499 |
| C | 7.741726 | 5.967107 | 3.916705 |
| H | 7.828765 | 4.360447 | 5.35423 |
| H | 7.813616 | 7.364055 | 2.274359 |
| C | 13.27581 | 7.351515 | 1.486529 |
| H | 13.23856 | 7.651821 | -0.64971 |
| H | 13.1607 | 6.761904 | 3.558511 |
| C | 12.86553 | 1.39577 | -2.52505 |
| H | 14.72981 | 1.680111 | -1.47692 |
| H | 10.86935 | 1.307559 | -3.33838 |
| H | 7.167961 | 6.626492 | 4.562553 |
| H | 13.82869 | 8.26484 | 1.689813 |
| H | 13.31031 | 0.820207 | -3.33253 |
| C | 1.419883 | -3.17819 | -1.53478 |
| C | 1.244944 | -1.80149 | -1.30586 |
| C | 0.269911 | -3.96081 | -1.73212 |
| C | -0.02689 | -1.23794 | -1.28738 |
| H | 2.114705 | -1.17281 | -1.15225 |
| C | -1.0025 | -3.39754 | -1.69493 |
| H | 0.375558 | -5.02876 | -1.90392 |
| C | -1.18002 | -2.02394 | -1.46335 |
| H | -0.13266 | -0.16766 | -1.13293 |
| H | -1.87278 | -4.02891 | -1.84488 |
| C | -2.5379 | -1.40242 | -1.46815 |
| C | -3.58615 | -1.91211 | -0.75375 |
| C | -2.66157 | -0.17811 | -2.31751 |
| C | -4.99751 | -1.47405 | -0.96788 |
| C | -3.40715 | -2.9532 | 0.304326 |
| C | -2.15592 | -0.16289 | -3.62937 |
| C | -3.22845 | 1.004379 | -1.81222 |
| C | -5.82854 | -1.15811 | 0.122146 |
| C | -5.56122 | -1.41938 | -2.25287 |
| C | -4.2375 | -4.08723 | 0.341675 |
| C | -2.44958 | -2.79916 | 1.321405 |
| C | -2.24389 | 0.982637 | -4.41887 |
| H | -1.69508 | -1.06104 | -4.03102 |
| C | -3.30463 | 2.154172 | -2.59676 |
| H | -3.60777 | 1.017246 | -0.7951 |
| C | -7.15202 | -0.771 | -0.06282 |
| H | -5.42695 | -1.21736 | 1.130054 |
| C | -6.88995 | -1.04889 | -2.43882 |
| H | -4.94864 | -1.66761 | -3.11398 |
| C | -4.09247 | -5.04959 | 1.339791 |
| H | -4.99824 | -4.21355 | -0.42361 |
| C | -2.31271 | -3.75483 | 2.327075 |
| H | -1.81293 | -1.9198 | 1.320236 |
| C | -2.81822 | 2.147356 | -3.90557 |
| H | -1.85798 | 0.966829 | -5.43495 |
| H | -3.7389 | 3.060358 | -2.1815 |
| C | -7.71341 | -0.7132 | -1.35106 |
| H | -7.76433 | -0.51922 | 0.795642 |
| H | -7.29565 | -1.00613 | -3.44611 |
| C | -3.12899 | -4.88757 | 2.337533 |
| H | -4.73678 | -5.92506 | 1.340846 |
| H | -1.57122 | -3.61015 | 3.109056 |
| H | -2.87931 | 3.043545 | -4.51729 |
| H | -3.02091 | -5.6334 | 3.120621 |
| C | -9.14378 | -0.37066 | -1.5706 |
| C | -9.91522 | -1.14291 | -2.419 |
| C | -9.77052 | 0.738589 | -0.91471 |
| C | -11.2902 | -0.88352 | -2.63693 |
| H | -9.46575 | -2.00197 | -2.91097 |
| C | -11.1109 | 1.038973 | -1.09877 |
| O | -9.0302 | 1.512929 | -0.04643 |
| C | -11.9086 | 0.215448 | -1.95794 |
| C | -12.0744 | -1.70034 | -3.49587 |
| C | -11.7101 | 2.212076 | -0.38807 |
| C | -8.22277 | 2.530775 | -0.66418 |
| C | -13.2965 | 0.442506 | -2.1669 |
| C | -13.4146 | -1.44959 | -3.68097 |
| H | -11.5925 | -2.53228 | -4.00453 |
| C | -11.9298 | 3.453552 | -1.05895 |
| C | -12.0579 | 2.09291 | 0.956071 |
| C | -7.4564 | 3.250684 | 0.435437 |
| H | -8.87392 | 3.22701 | -1.21012 |
| H | -7.53263 | 2.069942 | -1.38414 |
| C | -14.0292 | -0.36758 | -3.00639 |
| H | -13.7758 | 1.26823 | -1.65165 |
| H | -14.0039 | -2.08093 | -4.34064 |
| C | -11.5943 | 3.650171 | -2.4312 |
| C | -12.5038 | 4.5589 | -0.3443 |
| C | -12.6241 | 3.186649 | 1.660291 |
| O | -11.8335 | 0.87879 | 1.533234 |
| C | -6.58978 | 4.395826 | -0.10319 |
| H | -8.17773 | 3.636065 | 1.167723 |
| H | -6.82965 | 2.519053 | 0.962043 |
| H | -15.0892 | -0.17613 | -3.1515 |
| C | -11.8143 | 4.85896 | -3.05159 |
| H | -11.1623 | 2.825231 | -2.98789 |
| C | -12.7181 | 5.792426 | -1.01707 |
| C | -12.8379 | 4.385283 | 1.019934 |
| H | -12.8919 | 3.082206 | 2.705107 |
| C | -12.1077 | 0.691359 | 2.919822 |
| C | -5.79659 | 5.119557 | 0.992513 |
| H | -5.89056 | 4.002814 | -0.8569 |
| H | -7.22697 | 5.121965 | -0.62875 |
| C | -12.383 | 5.945334 | -2.34187 |
| H | -11.5505 | 4.981649 | -4.09888 |
| H | -13.1544 | 6.617956 | -0.45879 |
| H | -13.2733 | 5.219102 | 1.56577 |
| C | -11.7123 | -0.73542 | 3.276879 |
| H | -13.1767 | 0.858231 | 3.12013 |
| H | -11.5344 | 1.414007 | 3.518868 |
| H | -6.49466 | 5.505882 | 1.750015 |
| H | -5.15376 | 4.395053 | 1.514252 |
| C | -4.93461 | 6.273393 | 0.464993 |
| H | -12.5516 | 6.893077 | -2.8458 |
| C | -11.9641 | -1.06354 | 4.753803 |
| H | -12.2744 | -1.4251 | 2.634386 |
| H | -10.6513 | -0.87403 | 3.033335 |
| C | -4.14241 | 6.987856 | 1.564617 |
| H | -5.57847 | 6.998577 | -0.05267 |
| H | -4.2391 | 5.888628 | -0.29491 |
| C | -11.5668 | -2.49786 | 5.126759 |
| H | -11.4071 | -0.35801 | 5.388082 |
| H | -13.0279 | -0.91038 | 4.988911 |
| H | -3.53854 | 7.806139 | 1.155994 |
| H | -4.81169 | 7.414043 | 2.322271 |
| H | -3.4626 | 6.29577 | 2.076802 |
| H | -12.1229 | -3.20446 | 4.493219 |
| H | -10.5034 | -2.65211 | 4.891916 |
| C | -11.814 | -2.8369 | 6.602075 |
| C | -11.4124 | -4.26983 | 6.966616 |
| H | -12.8773 | -2.68378 | 6.835988 |
| H | -11.2591 | -2.1291 | 7.234325 |
| H | -11.6026 | -4.48192 | 8.024913 |
| H | -11.9746 | -5.0018 | 6.373694 |
| H | -10.3456 | -4.44237 | 6.778654 |

## **Supplementary Table 4 |** Cartesian coordinates of optimized P-**3** (two repeating units) in the ground state calculated by the DFT, B3LYP/6-31G(d), Gaussian 09 program.

|  | X | Y | Zz |
| --- | --- | --- | --- |
| C | 10.05764 | -0.58834 | 1.524655 |
| C | 9.751241 | -1.36534 | 2.61608 |
| C | 10.05631 | -0.94044 | 3.939394 |
| C | 10.73768 | 0.314093 | 4.087911 |
| C | 11.02431 | 1.096545 | 2.940013 |
| C | 9.760642 | -1.72502 | 5.105227 |
| C | 10.25169 | -1.28617 | 6.328154 |
| C | 10.91725 | -0.05138 | 6.480765 |
| C | 11.13328 | 0.744742 | 5.382495 |
| C | 9.015663 | -3.01513 | 5.074289 |
| C | 7.715004 | -3.17879 | 4.488279 |
| C | 7.114641 | -4.48199 | 4.456858 |
| C | 7.801884 | -5.58163 | 5.036937 |
| C | 9.006537 | -5.39743 | 5.670628 |
| C | 9.591848 | -4.11387 | 5.699374 |
| C | 6.950481 | -2.0953 | 3.971907 |
| C | 5.70551 | -2.29227 | 3.424316 |
| C | 5.11873 | -3.58824 | 3.344086 |
| C | 5.836412 | -4.65081 | 3.865634 |
| C | 3.780829 | -3.77802 | 2.733849 |
| C | 3.359816 | -2.99502 | 1.644978 |
| C | 2.108046 | -3.17541 | 1.065803 |
| C | 1.215354 | -4.14769 | 1.547823 |
| C | 1.627559 | -4.91576 | 2.651628 |
| C | 2.882454 | -4.74126 | 3.224958 |
| C | -0.14713 | -4.32285 | 0.963325 |
| C | -1.25072 | -4.41499 | 1.968611 |
| C | -0.37197 | -4.39871 | -0.38355 |
| C | 0.730551 | -4.58206 | -1.37683 |
| C | -1.73845 | -4.31151 | -0.97898 |
| C | 0.79962 | -3.77178 | -2.52397 |
| C | 1.810602 | -3.94929 | -3.46713 |
| C | 2.762176 | -4.9569 | -3.29562 |
| C | 2.693051 | -5.78421 | -2.17304 |
| C | 1.688713 | -5.59857 | -1.22416 |
| C | -2.18951 | -5.4596 | 1.924907 |
| C | -3.19507 | -5.55974 | 2.885216 |
| C | -3.28512 | -4.61639 | 3.910534 |
| C | -2.35302 | -3.57837 | 3.973799 |
| C | -1.3407 | -3.48561 | 3.019896 |
| C | -2.14885 | -5.21841 | -1.97228 |
| C | -3.41268 | -5.13589 | -2.54702 |
| C | -4.31872 | -4.12887 | -2.17163 |
| C | -3.8971 | -3.20416 | -1.20101 |
| C | -2.63795 | -3.29458 | -0.61651 |
| O | 10.03713 | -2.05138 | 7.462298 |
| O | 10.81837 | -3.97666 | 6.327094 |
| C | -5.66956 | -4.04253 | -2.7768 |
| C | -6.26666 | -2.77849 | -3.05148 |
| C | -7.52582 | -2.67731 | -3.59306 |
| C | -8.29658 | -3.83281 | -3.90261 |
| C | -7.68627 | -5.11189 | -3.67513 |
| C | -6.39103 | -5.17826 | -3.10075 |
| C | -9.61538 | -3.771 | -4.46766 |
| C | -10.2026 | -4.95931 | -4.88353 |
| C | -9.60753 | -6.21936 | -4.6615 |
| C | -8.38262 | -6.29355 | -4.04446 |
| C | -10.3687 | -2.50472 | -4.69003 |
| C | -10.6299 | -1.53464 | -3.66384 |
| C | -11.3159 | -0.31961 | -4.00039 |
| C | -11.755 | -0.11257 | -5.33536 |
| C | -11.5739 | -1.08373 | -6.28973 |
| C | -10.8998 | -2.27667 | -5.95323 |
| C | -10.2847 | -1.73302 | -2.29774 |
| C | -10.5511 | -0.77939 | -1.34475 |
| C | -11.1874 | 0.451811 | -1.6752 |
| C | -11.5602 | 0.64922 | -2.9933 |
| C | -11.4422 | 1.475031 | -0.63282 |
| C | -10.5232 | 1.692016 | 0.408053 |
| C | -10.7501 | 2.659351 | 1.381603 |
| C | -11.9099 | 3.452013 | 1.363635 |
| C | -12.8419 | 3.217389 | 0.337145 |
| C | -12.6087 | 2.259114 | -0.64394 |
| C | -12.1819 | 4.461751 | 2.429931 |
| C | -13.5518 | 4.389204 | 3.02498 |
| C | -11.2499 | 5.377192 | 2.83266 |
| C | -11.408 | 6.201197 | 4.069988 |
| C | -9.99056 | 5.633782 | 2.069039 |
| C | -14.0944 | 3.153436 | 3.419556 |
| C | -15.3777 | 3.072929 | 3.958075 |
| C | -16.1563 | 4.224293 | 4.092569 |
| C | -15.6397 | 5.455347 | 3.683781 |
| C | -14.352 | 5.536886 | 3.156423 |
| C | -11.1583 | 7.584864 | 4.043623 |
| C | -11.279 | 8.357454 | 5.197576 |
| C | -11.6296 | 7.759582 | 6.409911 |
| C | -11.8585 | 6.382976 | 6.45657 |
| C | -11.7495 | 5.612866 | 5.299814 |
| C | -8.75157 | 5.691326 | 2.731589 |
| C | -7.57551 | 5.95422 | 2.030974 |
| C | -7.61477 | 6.188776 | 0.654834 |
| C | -8.84047 | 6.157451 | -0.01301 |
| C | -10.0147 | 5.88238 | 0.686172 |
| O | -11.4488 | -4.92954 | -5.48653 |
| H | 9.839065 | -0.96261 | 0.528684 |
| H | 9.287317 | -2.33329 | 2.466557 |
| H | 11.50575 | 2.060082 | 3.087357 |
| H | 11.23757 | 0.244869 | 7.474781 |
| H | 11.63656 | 1.702568 | 5.487549 |
| H | 7.345468 | -6.5675 | 4.997739 |
| H | 9.534525 | -6.21758 | 6.147078 |
| H | 7.35097 | -1.09002 | 4.033825 |
| H | 5.139438 | -1.43407 | 3.074193 |
| H | 5.427389 | -5.65684 | 3.81684 |
| H | 4.029875 | -2.24703 | 1.230441 |
| H | 1.816413 | -2.55747 | 0.222573 |
| H | 0.949498 | -5.65514 | 3.06812 |
| H | 3.158693 | -5.33958 | 4.088664 |
| H | 0.055451 | -2.99385 | -2.67058 |
| H | 1.852114 | -3.30261 | -4.33983 |
| H | 3.546309 | -5.10079 | -4.03421 |
| H | 3.420808 | -6.57991 | -2.03639 |
| H | 1.6402 | -6.24584 | -0.35392 |
| H | -2.12509 | -6.19717 | 1.130904 |
| H | -3.90743 | -6.37909 | 2.833903 |
| H | -4.07018 | -4.69335 | 4.658082 |
| H | -2.41086 | -2.84109 | 4.770411 |
| H | -0.61159 | -2.68253 | 3.082502 |
| H | -1.46377 | -5.99591 | -2.29822 |
| H | -3.69207 | -5.8446 | -3.32164 |
| H | -4.57382 | -2.41644 | -0.88168 |
| H | -2.34578 | -2.57049 | 0.137453 |
| H | -5.69788 | -1.87354 | -2.85799 |
| H | -7.93408 | -1.69693 | -3.80957 |
| H | -5.9717 | -6.16115 | -2.90065 |
| H | -10.1441 | -7.10822 | -4.97838 |
| H | -7.9183 | -7.25839 | -3.85671 |
| H | -12.2627 | 0.815936 | -5.58431 |
| H | -11.927 | -0.95836 | -7.30856 |
| H | -9.82103 | -2.66658 | -2.00084 |
| H | -10.3006 | -0.98153 | -0.30738 |
| H | -12.0425 | 1.578549 | -3.28597 |
| H | -9.6059 | 1.110763 | 0.443287 |
| H | -10.0169 | 2.80929 | 2.167851 |
| H | -13.7633 | 3.792414 | 0.313434 |
| H | -13.3573 | 2.093008 | -1.41377 |
| H | -13.4989 | 2.251809 | 3.306805 |
| H | -15.7715 | 2.108571 | 4.268662 |
| H | -17.1598 | 4.161483 | 4.505245 |
| H | -16.242 | 6.355859 | 3.771888 |
| H | -13.9569 | 6.497393 | 2.840635 |
| H | -10.8722 | 8.054772 | 3.106816 |
| H | -11.0939 | 9.427561 | 5.150492 |
| H | -11.7164 | 8.359996 | 7.311639 |
| H | -12.119 | 5.905019 | 7.397385 |
| H | -11.9283 | 4.543109 | 5.343927 |
| H | -8.71501 | 5.523331 | 3.804354 |
| H | -6.62736 | 5.980698 | 2.561921 |
| H | -6.69918 | 6.401271 | 0.10929 |
| H | -8.88444 | 6.351571 | -1.08158 |
| H | -10.9645 | 5.861841 | 0.160711 |
| O | -10.7163 | -3.21969 | -6.95062 |
| C | -11.4683 | -4.40756 | -6.80022 |
| H | -12.53 | -4.24106 | -7.01668 |
| H | -11.026 | -5.10847 | -7.51778 |
| C | 10.79718 | -3.24121 | 7.5349 |
| H | 11.85153 | -3.0322 | 7.750414 |
| H | 10.33633 | -3.81563 | 8.346993 |
| C | 10.69646 | 0.677651 | 1.66228 |
| H | 10.9253 | 1.301031 | 0.823275 |

## **Supplementary Table 5 |** Cartesian coordinates of optimized P-**4** (two repeating units) in the ground state calculated by the DFT, B3LYP/6-31G(d), Gaussian 09 program.

|  | X | Y | Z |
| --- | --- | --- | --- |
| C | -13.3508 | -3.90525 | 1.318891 |
| C | -12.4737 | -4.88622 | 0.798103 |
| C | -11.2858 | -4.52207 | 0.203516 |
| C | -10.9065 | -3.15517 | 0.097797 |
| C | -11.7761 | -2.16704 | 0.666409 |
| C | -13.0039 | -2.57611 | 1.255494 |
| C | -9.67907 | -2.72249 | -0.50665 |
| C | -9.32803 | -1.38237 | -0.42428 |
| C | -10.1766 | -0.38414 | 0.13925 |
| C | -11.3919 | -0.80429 | 0.649952 |
| C | -8.71688 | -3.65417 | -1.16818 |
| C | -9.07425 | -4.50515 | -2.26711 |
| C | -8.07756 | -5.38376 | -2.80652 |
| C | -6.76605 | -5.3653 | -2.27296 |
| C | -6.39054 | -4.49615 | -1.26439 |
| C | -7.39816 | -3.63605 | -0.73687 |
| C | -10.3566 | -4.48919 | -2.88264 |
| C | -10.6489 | -5.32341 | -3.93896 |
| C | -9.67684 | -6.22191 | -4.43957 |
| C | -8.41874 | -6.24543 | -3.88495 |
| O | -8.09717 | -1.00116 | -0.93467 |
| O | -7.0552 | -2.74409 | 0.266925 |
| C | -9.7923 | 1.050527 | 0.189705 |
| C | -4.98745 | -4.46802 | -0.77537 |
| C | -10.0494 | 1.808657 | 1.344608 |
| C | -9.74494 | 3.165315 | 1.4016 |
| C | -9.17017 | 3.826012 | 0.303582 |
| C | -8.92845 | 3.069602 | -0.85727 |
| C | -9.21722 | 1.709979 | -0.91081 |
| C | -3.92323 | -4.51398 | -1.69147 |
| C | -2.59978 | -4.52782 | -1.26111 |
| C | -2.28317 | -4.50154 | 0.106688 |
| C | -3.34969 | -4.47668 | 1.02309 |
| C | -4.67254 | -4.44315 | 0.594598 |
| C | -8.88382 | 5.291457 | 0.333844 |
| C | -8.23022 | 5.897252 | 1.370174 |
| C | -9.37141 | 6.046478 | -0.86163 |
| C | -0.8681 | -4.55842 | 0.581424 |
| C | 0.107905 | -3.73814 | 0.088929 |
| C | -0.60461 | -5.58159 | 1.639326 |
| C | -0.19446 | -2.54308 | -0.7572 |
| C | 1.560285 | -3.96174 | 0.355579 |
| C | -8.18056 | 7.382341 | 1.535383 |
| C | -7.50603 | 5.127525 | 2.427507 |
| C | -8.52397 | 6.91542 | -1.56953 |
| C | -8.97405 | 7.587329 | -2.705 |
| C | -10.2824 | 7.406318 | -3.15755 |
| C | -11.1337 | 6.538396 | -2.47044 |
| C | -10.6793 | 5.857746 | -1.34202 |
| C | -1.08883 | -6.89433 | 1.500345 |
| C | -0.86931 | -7.84836 | 2.492709 |
| C | -0.1799 | -7.50464 | 3.657473 |
| C | 0.287661 | -6.19902 | 3.819622 |
| C | 0.078047 | -5.24856 | 2.821649 |
| C | 2.387984 | -2.90013 | 0.761306 |
| C | 3.747582 | -3.08899 | 0.985622 |
| C | 4.346964 | -4.34366 | 0.792796 |
| C | 3.524227 | -5.40003 | 0.368555 |
| C | 2.161147 | -5.21487 | 0.155804 |
| C | 0.518251 | -2.3097 | -1.94662 |
| C | 0.25915 | -1.18489 | -2.72827 |
| C | -0.70175 | -0.25508 | -2.32538 |
| C | -1.40104 | -0.46156 | -1.13464 |
| C | -1.15079 | -1.59311 | -0.3599 |
| C | -6.96476 | 8.029465 | 1.819061 |
| C | -6.91267 | 9.410633 | 1.999954 |
| C | -8.07976 | 10.17372 | 1.924241 |
| C | -9.29784 | 9.542564 | 1.664795 |
| C | -9.34745 | 8.162819 | 1.471986 |
| C | -7.69065 | 5.433322 | 3.787516 |
| C | -7.00097 | 4.737342 | 4.779053 |
| C | -6.09252 | 3.735285 | 4.43155 |
| C | -5.88146 | 3.436233 | 3.084147 |
| C | -6.58094 | 4.12381 | 2.093481 |
| C | 5.789436 | -4.59513 | 1.056203 |
| C | 6.167405 | -5.67596 | 1.831676 |
| C | 7.523582 | -5.96092 | 2.121623 |
| C | 8.552892 | -5.15323 | 1.537008 |
| C | 8.175167 | -4.09391 | 0.644661 |
| C | 6.826894 | -3.79097 | 0.494838 |
| C | 9.17389 | -3.28228 | -0.11244 |
| C | 10.15929 | -3.85918 | -0.98397 |
| C | 11.09913 | -2.98751 | -1.627 |
| C | 11.0113 | -1.58875 | -1.42352 |
| C | 9.099397 | -1.90165 | -0.01447 |
| C | 10.22401 | -5.24862 | -1.2814 |
| C | 11.18823 | -5.75113 | -2.12712 |
| C | 12.14399 | -4.89425 | -2.72243 |
| C | 12.09401 | -3.54193 | -2.47832 |
| C | 7.881322 | -7.01965 | 3.000541 |
| C | 9.197232 | -7.26129 | 3.318568 |
| C | 10.21362 | -6.4415 | 2.772794 |
| C | 9.901263 | -5.41762 | 1.906026 |
| H | -14.2884 | -4.20597 | 1.778418 |
| H | -12.7377 | -5.93774 | 0.872333 |
| H | -10.6204 | -5.28597 | -0.18192 |
| H | -13.6591 | -1.81326 | 1.669621 |
| H | -12.0792 | -0.06782 | 1.057937 |
| H | -6.03196 | -6.05683 | -2.67833 |
| H | -11.1087 | -3.79923 | -2.51793 |
| H | -11.6341 | -5.28725 | -4.396 |
| H | -9.92307 | -6.87998 | -5.26838 |
| H | -7.65586 | -6.91634 | -4.27305 |
| H | -10.4818 | 1.323484 | 2.215457 |
| H | -9.95199 | 3.722339 | 2.309874 |
| H | -8.50661 | 3.558056 | -1.73114 |
| H | -9.0087 | 1.154928 | -1.81843 |
| H | -4.13751 | -4.52221 | -2.75671 |
| H | -1.79819 | -4.55436 | -1.99265 |
| H | -3.1349 | -4.48064 | 2.088105 |
| H | -5.47148 | -4.41079 | 1.326934 |
| H | -7.50511 | 7.059985 | -1.22385 |
| H | -8.29902 | 8.250913 | -3.23929 |
| H | -10.6334 | 7.931311 | -4.04202 |
| H | -12.1529 | 6.386343 | -2.81654 |
| H | -11.343 | 5.173788 | -0.82037 |
| H | -1.63724 | -7.16564 | 0.60262 |
| H | -1.24211 | -8.8605 | 2.358027 |
| H | -0.01566 | -8.24568 | 4.43532 |
| H | 0.813206 | -5.91674 | 4.728274 |
| H | 0.442197 | -4.23477 | 2.955667 |
| H | 1.953701 | -1.91613 | 0.914444 |
| H | 4.344357 | -2.25566 | 1.340306 |
| H | 3.966839 | -6.37453 | 0.181251 |
| H | 1.553255 | -6.04936 | -0.17945 |
| H | 1.277021 | -3.02153 | -2.25956 |
| H | 0.812822 | -1.03221 | -3.65113 |
| H | -0.89849 | 0.626099 | -2.9303 |
| H | -2.14075 | 0.263356 | -0.80443 |
| H | -1.69717 | -1.74695 | 0.565476 |
| H | -6.05455 | 7.440842 | 1.892497 |
| H | -5.95932 | 9.8908 | 2.205326 |
| H | -10.2144 | 10.12489 | 1.616091 |
| H | -10.2981 | 7.678718 | 1.271621 |
| H | -8.38551 | 6.22122 | 4.064562 |
| H | -7.16965 | 4.98182 | 5.824679 |
| H | -5.5485 | 3.197619 | 5.203584 |
| H | -5.1661 | 2.667824 | 2.802229 |
| H | -6.41094 | 3.887431 | 1.047607 |
| H | 5.398048 | -6.30234 | 2.275724 |
| H | 11.74988 | -0.94865 | -1.89913 |
| H | 9.492936 | -5.91724 | -0.84259 |
| H | 11.2115 | -6.81582 | -2.34327 |
| H | 12.90444 | -5.30487 | -3.38102 |
| H | 12.8087 | -2.86893 | -2.94632 |
| H | 7.087923 | -7.6286 | 3.427602 |
| H | 9.458399 | -8.06927 | 3.996475 |
| H | 11.25073 | -6.61709 | 3.045717 |
| H | 10.69064 | -4.79126 | 1.506673 |
| H | -8.04119 | 11.2496 | 2.072964 |
| O | 6.471294 | -2.73075 | -0.31969 |
| O | 8.079121 | -1.3655 | 0.756059 |
| C | -6.98821 | -1.39247 | -0.14735 |
| H | -6.11454 | -1.20973 | -0.78223 |
| H | -6.93211 | -0.81611 | 0.782374 |
| C | 6.80583 | -1.43708 | 0.154139 |
| H | 6.738315 | -0.79163 | -0.72814 |
| H | 6.113944 | -1.10421 | 0.93592 |
| C | 10.01756 | -1.01573 | -0.65061 |
| H | 9.962452 | 0.048189 | -0.55087 |


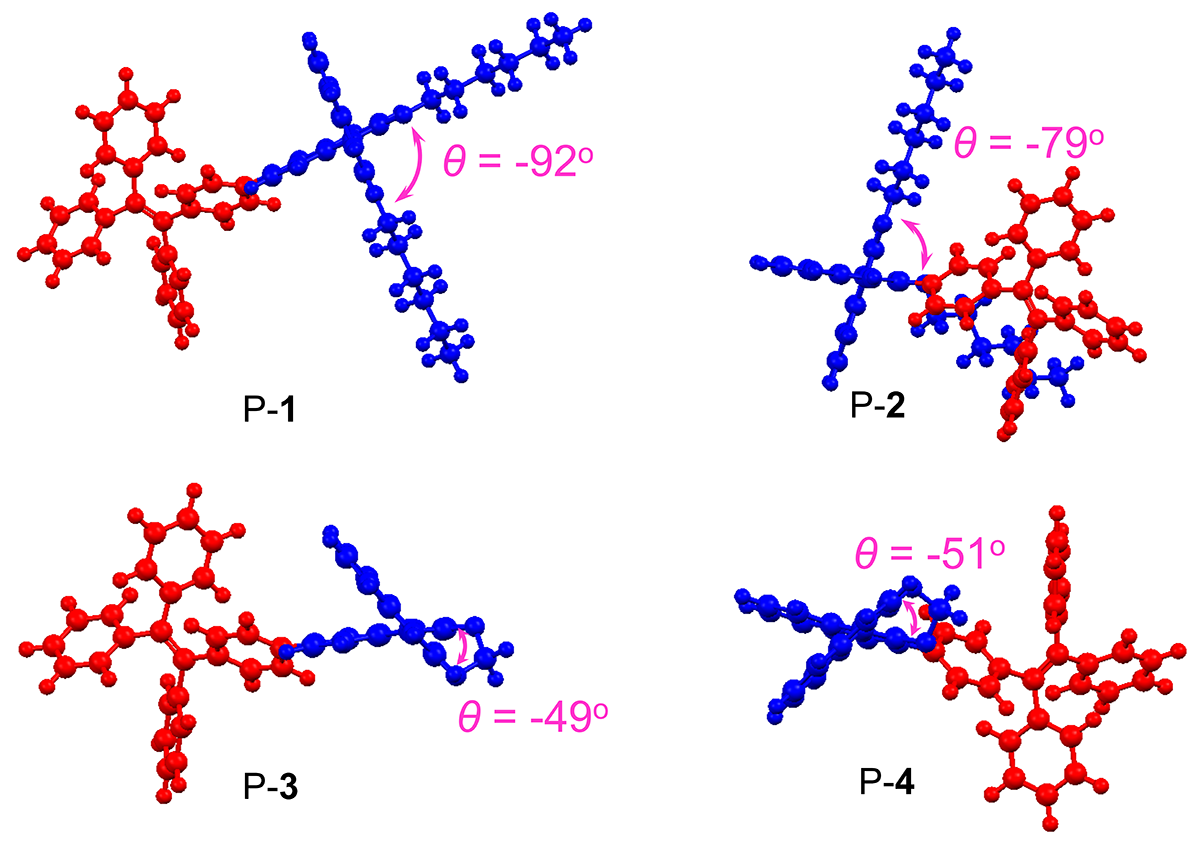


## **Supplementary Figure 9 |** Optimized structures of P-**1**, P-**2**, P-**3** and P-**4** in the ground state calculated by the DFT, B3LYP/6-31G(d), Gaussian 09 program. Only one repeating unit was drawn for clear presentation.

## **Supplementary Figure 10 |** (A and B) Simulated UV spectra of P-**2** with (A) varying *θ* from -70^o^ to -90^o^ and constant *β* (45^o^), and (B) *β* varying from 35^o^ to 55^o^ and constant *θ* (-80^o^). (C) Plots of *λ*_abs_ – (*λ*_abs_)_0_ versus *θ* and *β*. *λ*_abs_: simulated absorption maximum, (*λ*_abs_)_0_: simulated absorption maximum at *θ* = -80^o^ and *β* = 45^o^. Semiempirical CIS/ZINDO method was applied with NStates = 10, and all these calculations were performed using Gaussian 09 Program. For the sake of clear demonstration and simplified calculation, the hexyl groups were replaced with methyl groups in P-**2**.

## **Supplementary Figure 11 |** (A and B) Simulated UV spectra of P-**4** with (A) varying *θ* from -46^o^ to -56^o^ and constant *β* (49^o^), and (B) *β* varying from 44^o^ to 54^o^ and constant *θ* (-51^o^). (C) Plots of *λ*_abs_ – (*λ*_abs_)_0_ versus *θ* and *β*. *λ*_abs_: simulated absorption maximum, (*λ*_abs_)_0_: simulated absorption maximum at *θ* = -51^o^ and *β* = 49^o^. Semiempirical CIS/ZINDO method was applied with NStates = 10.

## **Supplementary Figure 12 |** PL spectra of polymer P-**3** in THF/water mixtures with different water fractions (*f*_w_). Concentration: 10^-5^ M, *λ*_ex_ = 345 nm.

## **Supplementary Figure 13 |** PL spectra of polymer P-**4** in THF/water mixtures with different water fractions (*f*_w_). Concentration: 10^-5^ M, *λ*_ex_ = 340 nm.

## **Supplementary Figure 14 |** Absolute fluorescence quantum yield of P-**1**, P-**2**, P-**3** and P-**4** in THF/water mixtures with different water fractions.

## **Supplementary Table 6 |** Summary of emission properties of polymer P-**1**, P-**2**, P-**3** and P-**4**.

| Polymer | Φ_F_ (%) | | |
| --- | --- | --- | --- |
|  | soln*^a^* | aggr*^b^* | film*^c^* |
| P-**1** | 1.3 | 41.8 | 59.7 |
| P-**2** | 0.7 | 24.5 | 19.2 |
| P-**3** | 2.0 | 35.8 | 48.2 |
| P-**4** | 2.4 | 29.2 | 34.2 |

*^α^*Fluorescence quantum yield in dilute THF solution. *^b^*Φ_F_ in THF/water mixture with *f*_w_ = 90%. *^c^*Φ_F_ in the film state.

## **Supplementary Figure 15 |** Distribution of dihedral angle *θ* of P-**1** in THF solution. A) to D) Representing four independent MD trajectories.

## **Supplementary Figure 16 |** Distribution of dihedral angle *θ* of P-**1** aggregate in water solution. A) to F) Representing six independent MD trajectories.

## **Supplementary Figure 17 |** Distribution of dihedral angle *θ* of P-**3** in THF solution. A) to D) Representing four independent MD trajectories.

## **Supplementary Figure 18 |** Distribution of dihedral angle *θ* of P-**3** aggregate in water solution. A) to F) Representing six independent MD trajectories.

## **Supplementary Figure 19 |** The calculated Gibbs free energies as a function of the representive dihedral angle *θ* in various conformations of A) single P-**1** in THF solution, B) P-**1** aggregate in water solution, C) single P-**3** in THF solution and D) P-**3** aggregate in water solution from molecular dynamics simulations.

## **Supplementary Figure 20 |** Distribution of dihedral angle *β* of P-**1** in THF solution. A) to D) Representing four independent MD trajectories.

## **Supplementary Figure 21 |** Distribution of dihedral angle *β* of P-**1** aggregate in water solution. A) to F) Representing six independent MD trajectories.

## **Supplementary Figure 22 |** Distribution of dihedral angle *β* of P-**3** in THF solution. A) to D) Representing four independent MD trajectories.

## **Supplementary Figure 23 |** Distribution of dihedral angle *β* of P-**3** aggregate in water solution. A) to F) Representing six independent MD trajectories.

##

**Supplementary Figure 24 |** A) and B) showed the relative Boltzmann populations of torsion angle *β* in various conformers of P-**1** in THF and water, respectively. C) and D) showed the relative Boltzmann populations of *β* in various conformers of P-**3** in THF and water, respectively.

# Supplementary References

1. Hu, R. et al. Luminogenic materials constructed from tetraphenylethene building blocks: Synthesis, aggregation-induced emission, two-photon absorption, light refraction, and explosive detection. *J. Mater. Chem.* **22**, 232-240 (2012).

2. Zhang, H. et al. Axial chiral aggregation-induced emission luminogens with aggregation-annihilated circular dichroism effect. *J. Mater. Chem. C* **3**, 5162-5166 (2015).

3. Yao, B. et al. Catalyst-Free Thiol–Yne Click Polymerization: A Powerful and Facile Tool for Preparation of Functional Poly(vinylene sulfide)s. *Macromolecules* **47**, 1325-1333 (2014).
